# Preliminary study of gut microbiome influence on Black Ivory Coffee fermentation in Asian elephants

**DOI:** 10.1038/s41598-025-24196-0

**Published:** 2025-11-18

**Authors:** Nodoka Chiba, Vachiranee Limviphuvadh, Chong Han Ng, Ryuto Koyagi, Yuta Kino, Yuya Nakamura, Takuji Yamada

**Affiliations:** 1 School of Life Science and Technology, Institute of Science Tokyo, Tokyo, 152-8550 Japan; 2 Biomolecular Sequence to Function Division (BSFD), Bioinformatics Institute (BII), Agency for Science, Technology and Research (A*STAR), 30 Biopolis Street, #07-01 Matrix, Singapore, 138671 Singapore; 3 Faculty of Information Science & Technology, Multimedia University, Jalan Ayer Keroh Lama, 75450 Melaka, Malaysia; 4 Metagen, Inc, Yamagata, Japan; 5 Metagen Therapeutics, Inc, Yamagata, Japan; 6 digzyme, Inc, Tokyo, Japan

**Keywords:** Black Ivory Coffee, Elephant dung coffee, Coffee, Gut microbiome, Fermentation, Metabolism, 16S rRNA gene sequencing analysis, Bacteria, Next-generation sequencing

## Abstract

Black Ivory Coffee (BIC), produced through the digestion of Arabica coffee beans by Asian elephants, is recognized for its smooth, chocolaty flavor and low bitterness. However, the biological mechanisms underlying its unique taste remain poorly understood. In this study, we conducted a preliminary analysis of the gut microbiome of BIC-producing elephants and compared it with that of control elephants. 16S rRNA gene sequencing analysis revealed significant differences in microbial community structure, including the enrichment of specific bacterial genera such as *Acinetobacter* and family such as Izemoplasmataceae in the BIC group. Functional prediction using PICRUSt2 indicated that the BIC elephant gut microbiome harbors key enzymes involved in the degradation and utilization of pectin and cellulose—major components of coffee cherries and beans. Notably, genes related to pectin transport and downstream metabolism, including K08191 and K01812, were significantly more abundant in BIC elephants. These pathways may contribute to the degradation of specific compounds, such as 2-furfuryl furan, which has been previously shown to decrease after gastrointestinal passage. Comparative analysis with other animals revealed that only elephants harbored a complete set of pectin-degrading genes. Our findings suggest that the gut microbiota of BIC elephants facilitates microbial fermentation of coffee components, potentially influencing the chemical profiles of Black Ivory Coffee.

**Supplementary Information:**

The online version contains supplementary material available at 10.1038/s41598-025-24196-0.

## Introduction

Coffee is one of the most consumed beverages in the world. The taste of coffee varies greatly depending on multiple factors, such as the type of coffee beans, roasting and extraction methods, and the processing of coffee cherries. There are several methods for processing coffee cherries, including the washed (wet) process, the natural (dry) process, or the semi-dry process^1^. Interestingly, certain types of coffee cherries are processed by passing through the digestive tract of animals. For example, *kopi luwak* is produced after coffee beans are passed through the intestinal tract of the civet cat in Indonesia, resulting in a flavor that is often described as less bitter than that of regular coffee^2^. Black Ivory Coffee (BIC), another example, is produced after coffee beans are passed through the intestinal tract of Asian elephants from specific farms in northeast Thailand. This coffee is said to have a chocolaty flavor and a less bitter taste than regular coffee^3^. Various chemical changes have been identified in these animal-produced coffees, including protein degradation and the formation of unique volatile components found in the beans after passing through the digestive tract^4,5^. However, the mechanism underlying these chemical transformations remains poorly understood.

It has been suggested that the chemical changes of the *kopi luwak* may result from fermentation by the gut microbiome^3^. Our previous study revealed that *Gluconobacter* was the dominant genus in the gut of civet cats and it may produce volatile compounds from the coffee beans, suggesting that microbial metabolism contributes to the coffee aroma^6^. Another study identified two bacterial strains, *Lactiplantibacillus plantarum* and *Paenibacillus motobuensis,* isolated from the feces of civet cats, which may be necessary for the fermentation of *kopi luwak*^7^. These findings raised the question of whether the gut microbiome of elephants similarly influences the flavor of BIC.

Here, we analyzed the gut microbial 16S rRNA gene amplicon sequence data from feces of Asian elephants that had digested coffee beans (BIC elephants), aiming to determine whether their gut microbiome is involved in modifying the chemical components of Arabica coffee beans in this study. The results suggest that the gut microbial composition of BIC elephants differs from that of the control elephants that did not consume the coffee beans. Notably, *Acinetobacter* and other bacterial taxa were significantly more abundant or potentially uniquely present in the gut of BIC elephants; interestingly, *Acinetobacter* has also been detected on the surface of coffee beans^8^. This suggests that ingestion of coffee beans may lead to the colonization of specific microbes in the gut of elephants. Furthermore, our findings indicate the presence of microbial proteins associated with metabolic pathways involved in pectin and cellulose degradation—key components of coffee cherries and beans.

Collectively, our results suggest that the ingestion of coffee beans alters the gut microbiome of BIC elephants and that this altered microbiome may contribute to the unique flavor of BIC through microbial fermentation, although this requires experimental confirmation.

## Results

### The community structure of the gut microbiome in BIC elephants differs from that of control Asian elephants

We analyzed gut microbial 16S rRNA gene amplicon sequencing data obtained from the feces of Asian elephants, both with and without the consumption of Arabica coffee beans. The average number of raw reads per sample was 29,780 (method D), 40,679 (method E), and 38,845 (method X), depending on the DNA extraction method. Taxonomic profiles of the gut microbiota were generated using QIIME2^9^. After quality control, the average number of high-quality reads per sample was 19,927 (Method D), 27,268 (Method E), and 25,812 (Method X). Following taxonomic assignment, an average of 24,336 reads per sample was classified, and a total of 214 bacterial genera were identified across all the samples.

**Fig. 1:**
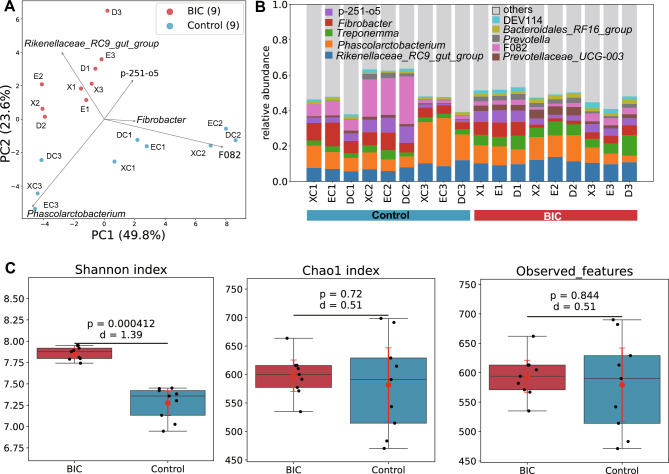
The community structure of the gut microbiome of elephants. (**A**) PCA plot of the gut microbial taxon profile. Red dots represent the samples of BIC elephants and blue dots represent the samples of control elephants. (**B**) The stacked bar plot of the relative abundance of genera of the gut microbiome. The top 10 genera with the highest average relative abundance are shown. Each bar plot represents the abundance of genera from each sample from the control elephant (left side) and the BIC elephant (right side). (**C**) Alpha diversity of the gut microbiome of the BIC and control elephants. *P* values were calculated by the two-tailed permuted Brunner–Munzel test. Cohen’s d and 95% confidence intervals were also calculated. 95% confidence intervals were shown in the box plots with red lines.

To assess differences in gut microbial community structure between the BIC and control elephant groups, we performed principal components analysis (PCA) and calculated alpha diversity indices. The result of PCA indicated differences in microbial community composition between the groups (Fig. 1A). The genera *Rikenellaceae_RC9_gut_group* and *Phascolarctobacterium* were predominant in both the BIC and the control elephant (Fig. 1B). The Shannon index, a measure of alpha diversity, was significantly higher in the BIC group (Fig. 1C, P = 0.000412, d = 1.39), while the Chao1 index and the number of observed features did not differ significantly between groups (*P* = 0.72 and 0.844, d = 0.51). These results suggest that the BIC elephants possess a more even distribution or richness in relatively abundant bacterial taxa. We also performed a permutational multivariate analysis of variance (PERMANOVA), which showed that classification (BIC vs. Control) (p.adj = 0.0002), age (p.adj = 0.0002), sex (p.adj = 0.03033), and developmental stage (adult or child) (p.adj = 0.034263) significantly explained the differences in gut microbiota between groups, whereas the DNA extraction method did not (p.adj = 0.9991, Supplementary Table 1).

Collectively, these findings indicate that the gut microbiota of BIC elephants is distinct from that of control elephants in terms of overall community structure.

### Specific gut microbiota are more abundant in BIC elephants

Next, we investigated differences in the relative abundance of individual bacterial genera between the BIC and control groups to characterize the gut microbiome of BIC elephants.

**Fig. 2:**
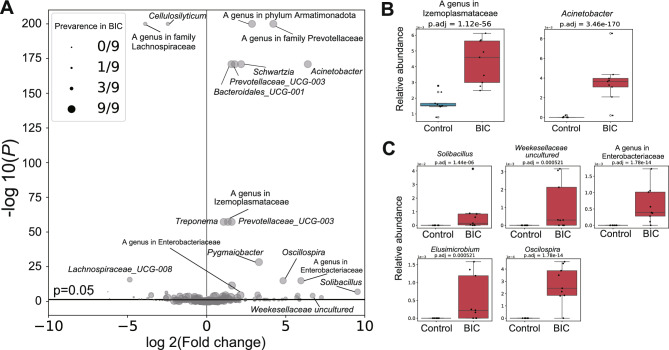
Differential analysis of the abundance of individual genera in gut microbiota. (**A**) Comparison of each bacterial abundance between BIC elephants and control elephants. X-axis, the ratio of the mean relative abundance of each group (fold change) transformed by -log_2_; Y-axis, the *P* value transformed by -log_10_. The horizontal line represents *P* = 0.05. *P* values were calculated by the two-tailed permuted Brunner–Munzel test. The plot size is each bacterium’s prevalence (number of samples) in the BIC elephants’ group. (**B**) The relative abundances of genera that were significantly higher in the BIC elephant group (red) than in the control elephant group (blue). *P* values were calculated by the two-tailed permuted Brunner–Munzel test and adjusted by Benjamini–Hochberg correction. (**C**) The relative abundances of genera that were detected from only the BIC elephant group (red, right) in this research. They were significantly higher in the BIC elephant group than in the control elephant group (left). *P* values were calculated by the two-tailed permuted Brunner–Munzel test and adjusted by Benjamini–Hochberg correction.

Differential abundance analysis revealed that 40 bacterial genera were significantly more abundant in the BIC group compared to the control group (*P* < 0.05). Of these, 24 genera were detected in more than six samples, corresponding to at least half the elephants in the BIC group (Fig. 2A, B**; **Supplementary Figure S1).

Furthermore, five bacterial genera were uniquely detected in at least six samples from the BIC group and not detected in any samples from the control group. These included *Solibacillus*, *Weeksellaceae,* a genus in Enterobacteriaceae, *Elusimicrobium*, and *Oscillospira* (Fig. 2C).

Taken together, these findings suggest that the gut microbiota of BIC elephants differs from that of the control elephants even at the genus level. Moreover, these changes in bacterial composition may be influenced by coffee bean consumption or dietary adaptation to coffee cherries.

### Several bacterial metabolic pathways in the gut of BIC elephants are associated with the degradation of coffee bean components

We next estimated the functional profiles of the gut microbiome using PICRUSt2 based on the taxon profiles generated by QIIME2. In total, 10,543 functional gene orthologs (KEGG orthologs; KOs) were detected across all samples.

To investigate whether the gut microbiome contributes to the flavor of BIC—which is characterized by chocolaty notes and low bitterness^3^—we focused on microbial metabolic functions. Degradation of pectin, cellulose, proteins, and carbohydrates, which are components of coffee cherries or beans, is known to reduce bitterness and contribute to a mild flavor^5^. We therefore examined whether the gut microbiome of BIC elephants possesses ortholog genes associated with the metabolism of these components.

Pectin degradation pathways by bacteria include several known pathways. For example, *Erwinia chrysanthemi* degrades pectin and imports its broken-down products via transporters such as the ABC transporter TogMNAB, electrochemical Potential-driven Transporter KdgT, and major facilitator superfamily (MFS) transporter ExuT^10^. We assessed whether the gut microbiome of BIC elephants contains the relevant KOs involved in pectin degradation, transport, and utilization.

**Fig. 3:**
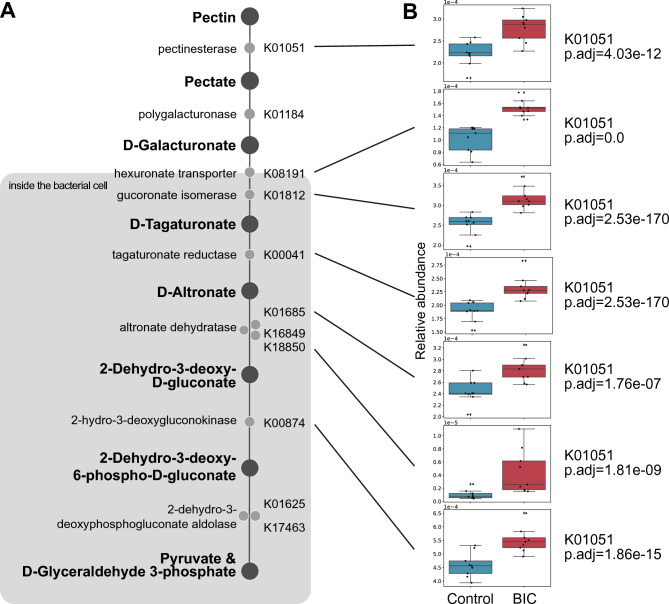
Pectin degradation pathway. (**A**) The pathway to degrade pectin to pyruvate and D-glyceraldehyde 3-phosphate. The KO definition is shown on the left side, and the KO ID is on the right. (**B**) KO abundance with the pectin degradation pathway. The box plot represents the relative abundance of the KOs. The relative abundances of the KO that were significantly higher in the BIC elephant group (red) than in the control elephant group (blue). *P* values were calculated by the two-tailed permuted Brunner–Munzel test and adjusted by Benjamini–Hochberg correction.

We first examined the presence of pectin esterase (PemA; K01051) and polygalacturonase (K01184), based on the previous report^11^ and the KEGG pathway^12^ (Fig. 3A). Several genera, including *Christensenellaceae_R-7_group* and *Clostridia_vadinBB60_group,* harbored these KOs. The abundance of K01051 was significantly higher in the BIC group (Supplementary Figure S2).

We next analyzed KOs related to transporters. Although the complete TogMNAB complex (K10192-K10195) was not detected, the KdgT transporter (K02526) and ExuT transporter (K08191) were present in the gut microbiome of elephants, and K08191 was significantly more abundant in the BIC group compared to controls (Supplementary Figure S3; *P* < 0.05, Fig. 3B).

To explore downstream catabolism, we examined eight KOs involved in the degradation of D-galacturonate following its import into bacterial cells: K01812, K00041, K01685, K16849, K18850, K00874, K01625, and K17463. These enzymes contribute to the conversion of D-galacturonate to intermediates such as D-Tagaturonate, D-Altronate, 2-dehydro-3-deoxy-D-gluconate, 2-Dehydro-3-deoxy-6-phospho-D-gluconate, and finally pyruvate and D-glyceraldehyde 3-phosphate **(**Fig. 3A**)**^11^. Among these, five KOs were significantly more abundant in the BIC group (*P* < 0.05, Fig. 3B).

We identified six bacterial genera and species—*Christensenellaceae*_R-7_group genera, *Clostridia*_vadinBB60_group genera, two types of species of *Candidatus Saccharimonas* genera, a species in the order of *Rhodospirillales*, and *Rickettsiales*—that harbored the relevant pectin degradation and utilization KOs (Supplementary Figure S3). These bacteria have previously been implicated in pectin utilization^13,14^. Collectively, these findings suggest that the gut microbiome of BIC elephants has the capacity to degrade pectin and assimilate its breakdown products, such as D-Galacturonate and 2-Dehydro-3-deoxy-D-gluconate.

To determine whether animals other than Asian elephants have the potential to ferment coffee beans via gut microbial pectin degradation, we analyzed the presence of pectin degradation-related KOs in the gut microbiomes of cattle, pigs, and chickens using large-scale gut microbial metagenomic-assembled genome data^15^. We selected these animals for comparison because: (1) cattle are known as herbivores like elephants, and Izemoplasmataceae, which was significantly more abundant in BIC elephants compared to the controls, has also been reported in cattle rumen^17^; (2) pigs’ gut microbiota composition is similar to that of elephants^16^; and (3) chickens may offer practical advantages for coffee production due to their short growth cycle.

We found that at least one bacterial taxon in each of the three reference animals carried K01051 and K01184. Furthermore, cattle and pigs harbored microbial taxa that possessed a full set of KOs related to D-galacturonate degradation (Supplementary Figure S4). However, these KO sets were not detected in chickens. In contrast, six distinct microbial taxa in the BIC elephant group harbored all 11 essential KOs required for complete pectin degradation. Based on these results, we propose that gut microbes in the Asian elephants—such as *Christensenellaceae_R-7_group* and *Saccharimonas*—may degrade pectin and metabolize its breakdown products to support their growth or activity.

Regarding cellulose metabolism, the gene encoding cellulose 1,4-beta-cellobiosidase (K19668), an enzyme responsible for converting cellulose into cellobiose, was significantly more abundant in the gut microbiome of BIC elephants (Supplementary Figure S5). These results suggest that the BIC elephant microbiota may degrade cellulose in coffee cherries and beans, contributing to the fermentation process by breaking down plant cell walls. The three reference animals also possess this KO, thus they may also degrade cellulose.

Overall, these findings demonstrate that the microbial environment of the BIC elephant is potentially well-suited for the degradation of pectin and cellulose, two key components contributing to bitterness in coffee. This microbial activity may reduce bitterness and contribute to BIC’s unique flavor. Furthermore, the elephant gut environment appears more capable of coffee fermentation than cattle, pigs, or chickens.

In total, 3,215 KOs were significantly more abundant and 651 KOs were significantly less abundant in the BIC group compared to the control group (*P* < 0.05). A summary of differential KO abundance is provided in Supplementary Figure S6.

## Discussion

This study is the first to analyze the gut microbiome of elephants that are involved in the production of Black Ivory Coffee, a premium and distinctive coffee product. Our findings provide preliminary insights into the relationship between the flavor characteristics of BIC and the gut microbiota of the elephants that produce it.

Several bacterial genera were significantly more abundant in BIC elephants compared to control elephants, and these alterations may be attributable to the ingestion and digestion of coffee beans. Notably, the relative abundance of *Acinetobacter* and a genus in Izemoplasmataceae was significantly higher in the BIC group. Among these, Izemoplasmataceae has been previously reported to be associated with pectin degradation. Although Izemoplasmataceae was reported in cattle rumen, Izemoplasmataceae, classified within the class Mollicutes of the phylum Tenericutes, includes specific species that have been suggested to utilize pectin, a major component of the coffee cherry^17^. *Acinetobacter* has been detected on the surface of coffee beans and inside coffee machines^8^, as well as in the intestinal tracts of several mammalian species^18^. It is suggested that these bacteria colonize the gut of BIC elephants after ingestion of coffee beans. The abundance of *Solibacillus* and *Weeksellaceae* have previously been reported to be higher in the gut of Asian elephants following habitat relocation and anthelmintic treatment^19^. A genus of Enterobacteriaceae was also found in the gut microbiome of civet cats that produce *kopi luwak*^6^. Additionally, *Oscillospira* was previously shown to be higher in Asian elephants when the diet shifted from milk to solid feed^20^. Taken together, these bacteria were related to environmental or dietary change.

Functional profile analysis revealed that the gut microbiome of BIC elephants harbors enzymes involved in the degradation and utilization of coffee bean components, particularly pectin and cellulose. In addition, the abundance of *Acinetobacter* was significantly higher in the BIC group, which is known to possess pectin lyase activity^21^. These results suggest that the gut microbiota is potentially capable of fermenting coffee components and may contribute to the modification of their chemical composition.

**Fig. 4:**
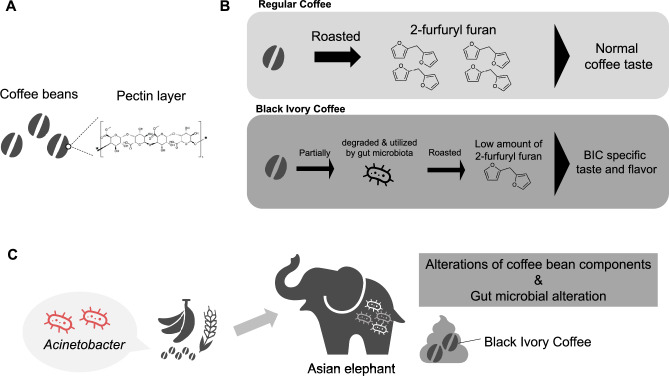
Hypothesis about the BIC fermentation by the gut microbiome of elephants. (**A**) Coffee beans are covered with a pectin layer. (**B**) Propose a mechanism of pectin conversion in regular coffee and BIC environments. (**C**) The overview of the hypothesis based on our results.

A previous study reported that the concentration of 2-furfuryl furan, a volatile compound known to form during the heating of pectin, was significantly reduced in roasted Arabica coffee beans after passage through the elephants^4,22^. Based on these studies and our results, we propose that the degradation and intake of pectin by the gut microbiome of BIC elephants may cause a decrease in the level of 2-furfuryl furan in the coffee beans, and it contributes to the unique flavor profile of Black Ivory Coffee. In other words, the gut microbiome of BIC elephants may contribute to the flavor by modifying the chemical composition, although further experimental validation is necessary (Fig. 4A, B).

Taken together, our results support a hypothesis in which coffee cherries ingested by elephants are enzymatically modified by the gut microbiome, leading to the development of taste and flavor characteristics of BIC **(**Fig. 4C**)**. Further experimental validation is required to test this hypothesis, such as biochemical analysis of coffee bean components before and after passage through the elephant digestive tract.

There are several limitations in this study. Firstly, the sample size is small, and age and sex were unbalanced. It reflects the limited availability of BIC production.

Although biological replicates were used, samples were obtained from only three elephants per group, which may limit statistical power and generalizability. Second, although certain genera were detected exclusively in the BIC group, this observation may reflect the detection threshold rather than true absence in controls, particularly given the limited sample size. Third, PICRUSt2 relies on inferred gene content from reference genomes, which may introduce inaccuracies, particularly in host-associated microbiomes with underrepresented taxa. Thus, the detection of rare taxa can be difficult, and the level of confidence in predictions is low. Fourth, there is a lack of direct chemical analysis of coffee beans before and after digestion.

Despite these limitations, our findings highlight a potential molecular mechanism by which the gut microbiota of BIC elephants contributes to the flavor of Black Ivory Coffee. This study provides a foundation for further exploration of animal-microbiome interactions in food fermentation and flavor development.

## Materials and methods

### Elephant fecal sample collection

Elephant fecal samples were collected on March 27, 2018, at approximately 8:00 am with the consent of Mr. Blake G. Dinkin, founder of Black Ivory Coffee, at his plantation in Ban Ta Klang Elephant Village, Surin Province, Thailand. A fecal sample was collected from each of the three BIC elephants and each of the three control elephants. The age and sex of each elephant are listed in Table 1. All elephants at Ban Ta Klang Elephant Village are housed under the same conditions and are typically fed grass. In addition, the BIC elephants received a snack made of mixed bananas, young and old rice bran, and coffee cherries at approximately 8:00 am on the day before sample collection. The control elephants were fed primarily grass with a small amount of sugarcane. All other conditions were identical for BIC and control elephants. In the control group, ID 1 and 3 elephants are parent and child.

**Table 1:** Characteristics of subjects.

Group	Individual ID	Age	Sex	DNA extraction method	Sequence ID
BIC	1	4	Male	D	D1
				E	E1
				X	X1
	2	17	Male	D	D2
				E	E2
				X	X2
	3	45	Male	D	D3
				E	E3
				X	X3
Control	1	15	Female	D	DC1
				E	EC1
				X	XC1
	2	17	Male	D	DC2
				E	EC2
				X	XC2
	3	1	Male	D	DC3
				E	EC3
				X	XC3

### DNA extraction and sequencing

Immediately after fecal collection, DNA was extracted using the QIAamp DNA Stool Kit (Qiagen, Germany). To optimize DNA yield and evaluate the robustness of microbial community recovery across different extraction protocols, each biological replicate from both the BIC and control groups was subjected to three distinct DNA extraction methods with slight modifications (Table 1):

- Method D: Standard QIAamp DNA Stool Kit protocol, following the manufacturer’s instructions without modification.
- Method E: Modified protocol incorporating a heating step at 80 °C, as recommended by the manufacturer for challenging samples.
- Method X: Modified protocol involving the addition of SDS and lysozyme to enhance lysis of Gram-positive and tough cell wall bacteria, based on the manufacturer’s guidance.

Following DNA extraction, the V3–V4 region of the 16S rRNA gene was amplified using primers 342 F and 806R. Amplicons were sequenced using paired-end 300 bp reads on the Illumina MiSeq platform.

### Taxon profiling

To estimate the taxon profile, QIIME2^9^ (2021.11 version) was used. The 3’ side of the raw reads were removed by the command “qiime dada2 denoise-paired” (–p-trim-left-f/r 5), and the 5’ side was trimmed by aligning the 240th base count from the 3’ side (–p-trunc-len-f/r 240). By default parameters of QIIME2, the chimeric reads were removed after denoising and merging the paired-end reads and their dereplication.

The command “qiime feature-classifier classify-sklearn” was used for taxon assignment and referenced silva-138–99-nb-classifier.qza, which is the full-length 16S rRNA gene information of the SILVA taxonomic reference database.

### Functional profiling

To estimate the functional profile, the QIIME2 2021.11 plugins PICRUSt2 and PICRUSt2^23^ (v2.5.2) were used. The input data of PICRUSt2 is a taxon profile, but our data includes many rare features. The distribution of reads per feature showed that the bottom 25% of read frequency was less than eight reads. Therefore, the command “qiime feature-table filter-features” (–p-min-frequency 8) was used to remove features with a total read frequency of fewer than eight reads per feature for the entire sample. Then, to estimate the abundance of each Kyoto Encyclopedia of Genes and Genomes (KEGG) orthologs (KEGG Orthology; KO)^24^ in each sample, we performed functional annotation using the command of the QIIME2 2021.11 plugins PICRUSt2 “qiime picrust2 full-pipeline” (–p-hsp method pic -p-max-nsti 2).

We downloaded the gut microbial genome data of mammals from MGnify^15^ on 14 April 2024 (https://www.ebi.ac.uk/metagenomics/browse/genomes). MGnify is a high-quality, comprehensive metagenome-assembled genome (MAG) database that covers various environmental sources. Following previous studies, we selected benchmark animals, so we used data from pigs, cattle, and chickens.

The proportion of bacteria contributing to each KO was determined by estimating the abundance of each KO using the command “picrust2_pipeline.py” (-m “pic”) in PICRUSt2 and calculating the nearest sequenced taxon index (NSTI) by the command “hsp.py” and “metagenome_pipeline.py” (-strat_out) in PICRUSt2.

### Statistical analysis

We used a two-tailed permutation-based Brunner–Munzel test^25^ to evaluate difference in bacterial abundance and KO abundance between the BIC group and the control group, as this test is suitable for two-group comparisons with fewer than ten samples. The significance threshold was set at *P* < 0.05. All the *P* values were adjusted by Benjamini–Hochberg correction in the differential analysis of bacterial abundance and KO abundance.

The mean fold change in abundance for each bacterium was calculated by adding 1.0 × 10^–5^ to the relative abundance values, allowing all differences between the groups to be visualized on the volcano plot. When the minimum abundance value in one group exceeded the maximum value in the other, the *P*-value was calculated as zero due to the mathematical properties of the permutation-based Brunner–Munzel test. To visualize the full distribution of bacteria or abundances in the volcano plot, such *P-*values were replaced with 1.0 × 10^–200^.

Principal component analysis (PCA) was performed to assess the separation of gut microbial composition between groups. The relative abundance values of each bacterium were standardized to z-scores. The Chao1 and Shannon indices were calculated to assess alpha diversity within each group. Independent t-tests were computed to evaluate between-group changes, using 95% confidence intervals, *P* values, and effect sizes for the primary outcome measures. Effect sizes (Cohen’s d) were also calculated to determine the magnitude of change for all significant post hoc outcomes.

## Supplementary Information

Supplementary Information 1.

Supplementary Information 2.

Supplementary Information 3.

Supplementary Information 4.

Supplementary Information 5.

Supplementary Information 6.

Supplementary Information 7.

Supplementary Information 8.

## Data Availability

The raw sequencing data reported in this paper have been deposited in the DNA Data Bank of Japan (DDBJ) Sequence Read Archive (DRA), Tokyo, Japan, under accession number PRJDB20573.
