# Supplementary figures and images for: Preliminary study of gut microbiome influence on Black Ivory Coffee fermentation in Asian elephants
Source: Sci Rep. 2025 Nov 18;15:40548. doi: 10.1038/s41598-025-24196-0 (PMC12627549; doi:10.1038/s41598-025-24196-0)

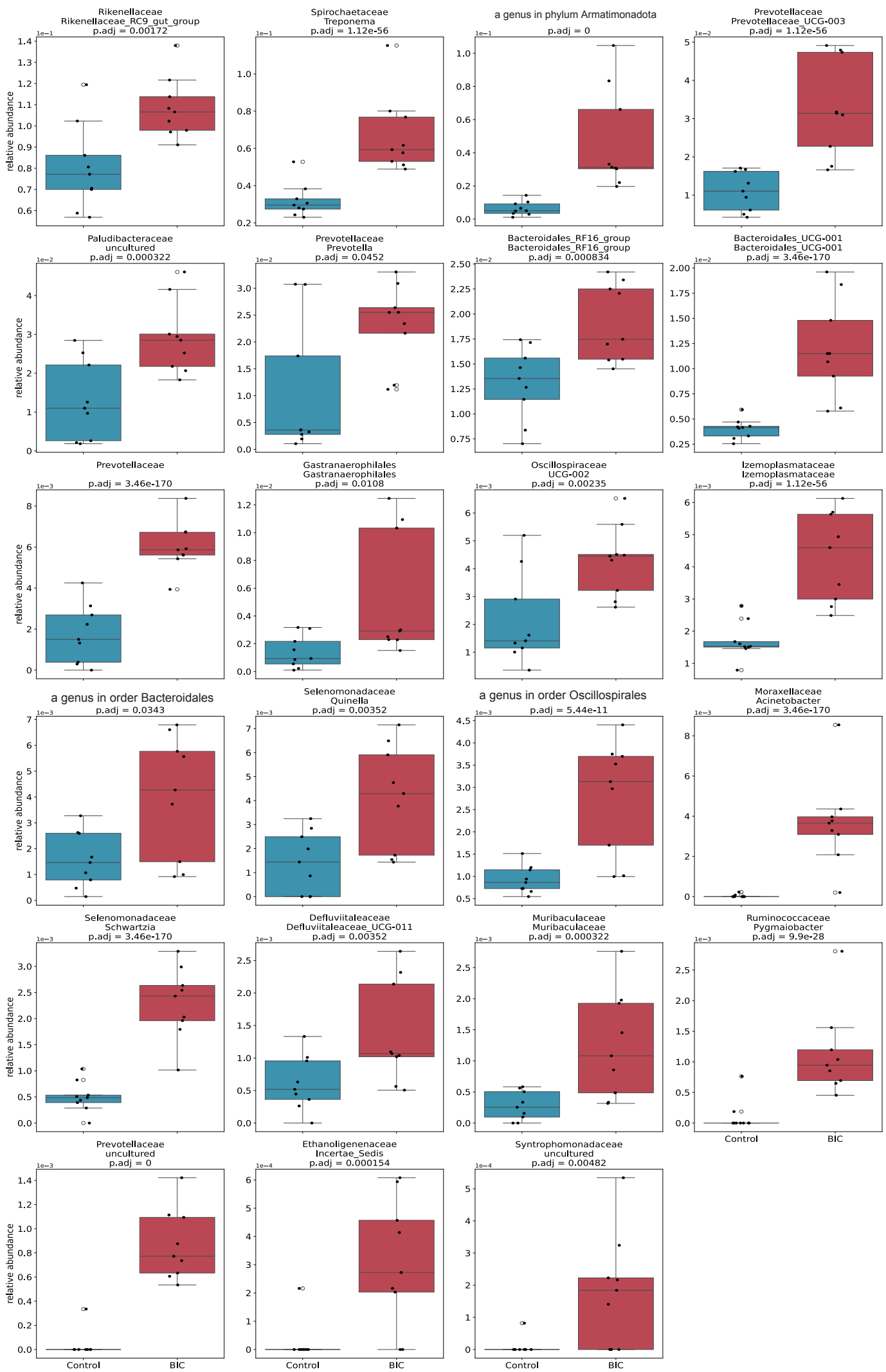

Supplement: Supplementary file 1 — Supplementary Information 1. [file 41598_2025_24196_MOESM1_ESM.pdf]

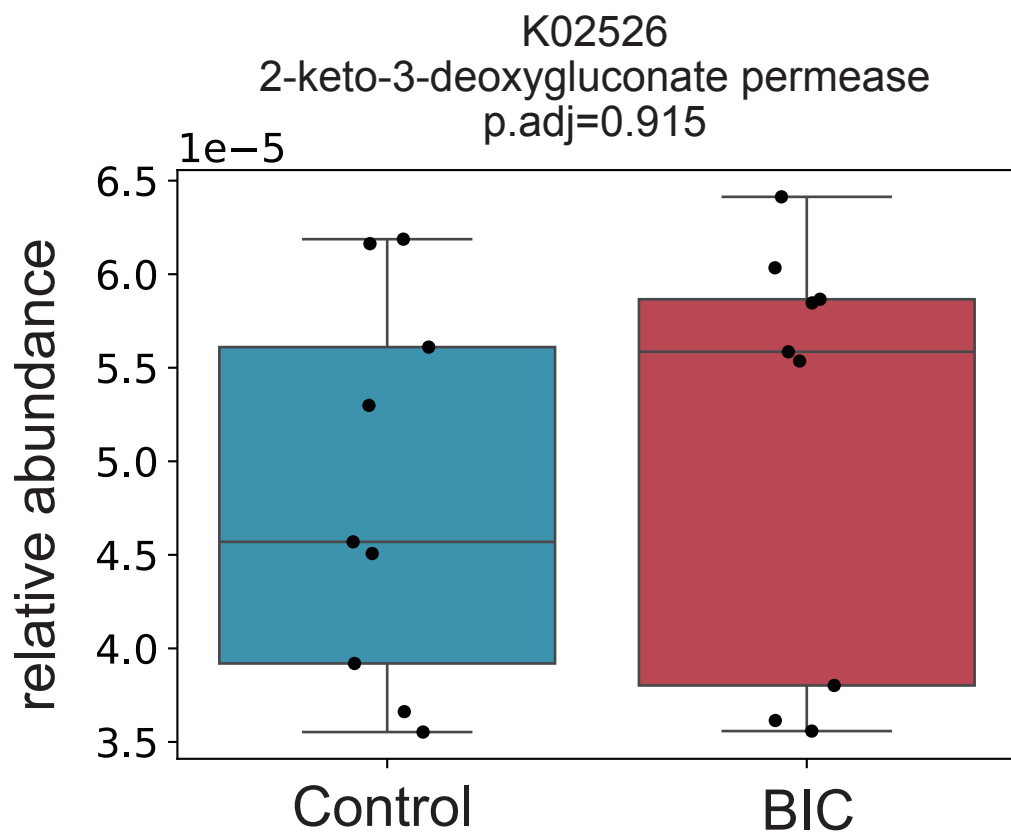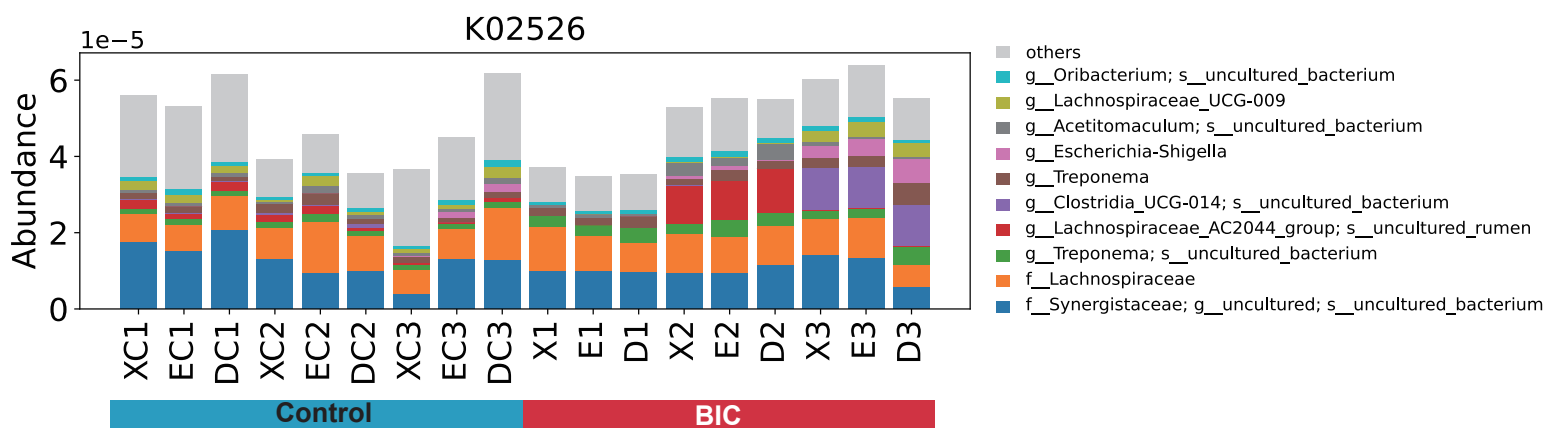

Supplement: Supplementary file 2 — Supplementary Information 2. [file 41598_2025_24196_MOESM2_ESM.pdf]

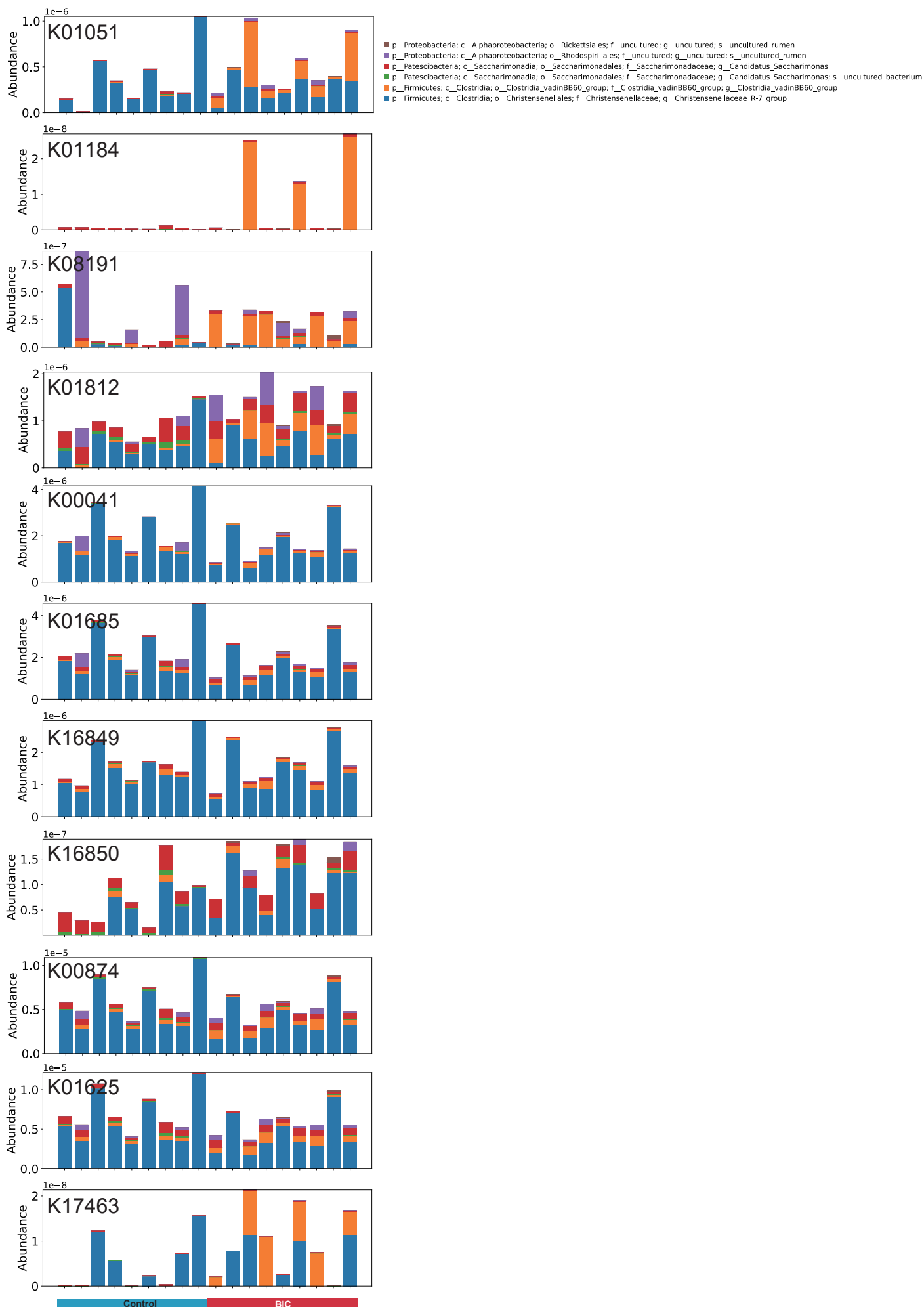

Supplement: Supplementary file 3 — Supplementary Information 3. [file 41598_2025_24196_MOESM3_ESM.pdf]

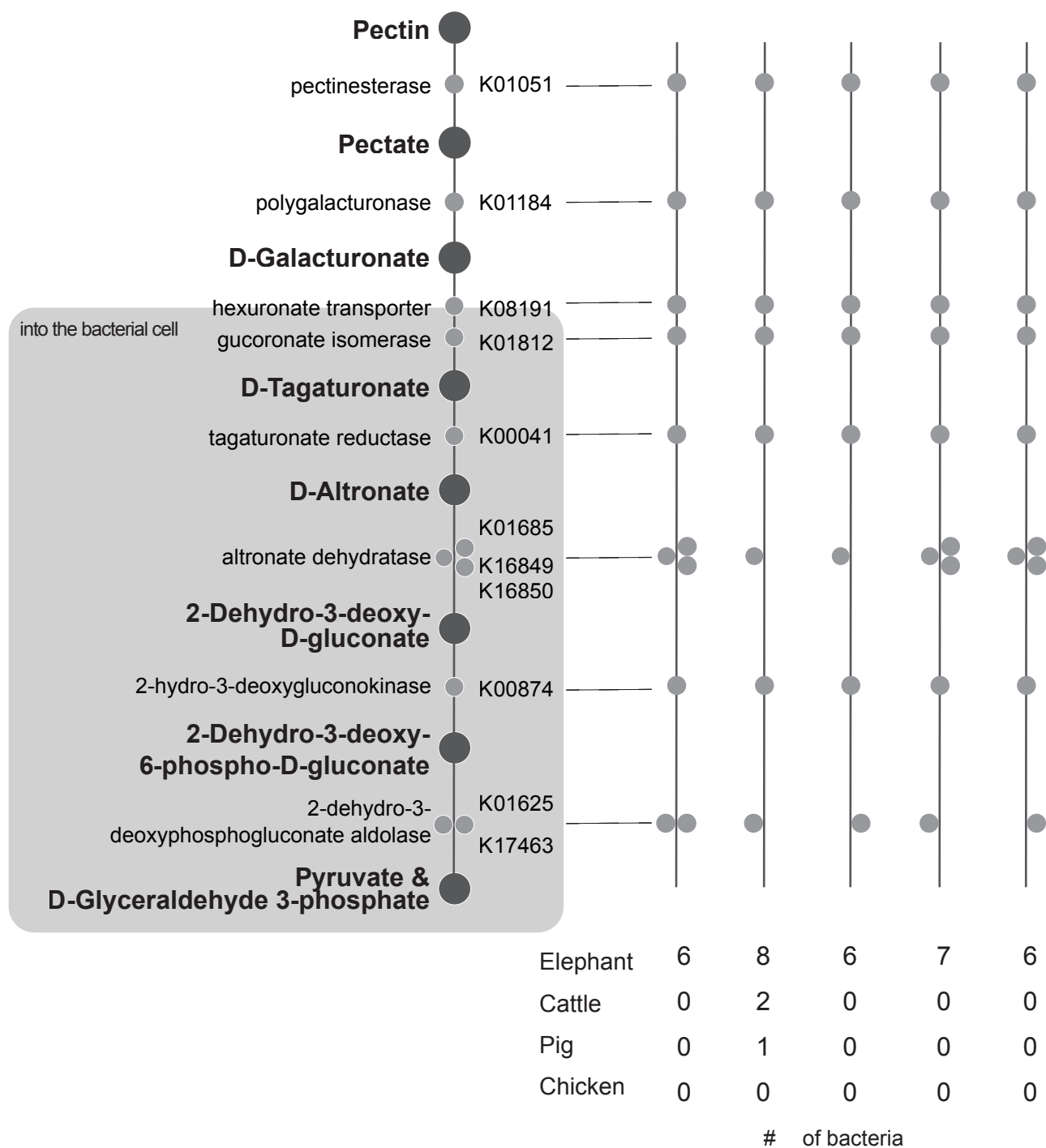

Supplement: Supplementary file 4 — Supplementary Information 4. [file 41598_2025_24196_MOESM4_ESM.pdf]

A

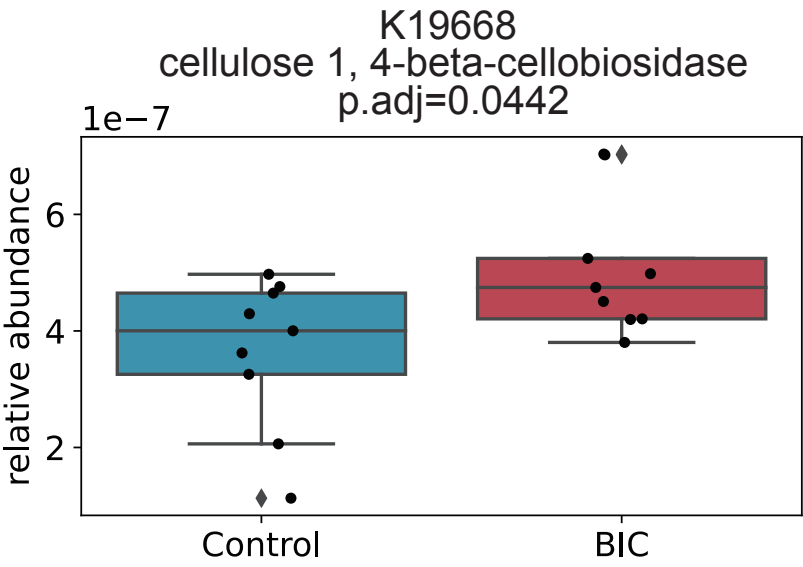

B

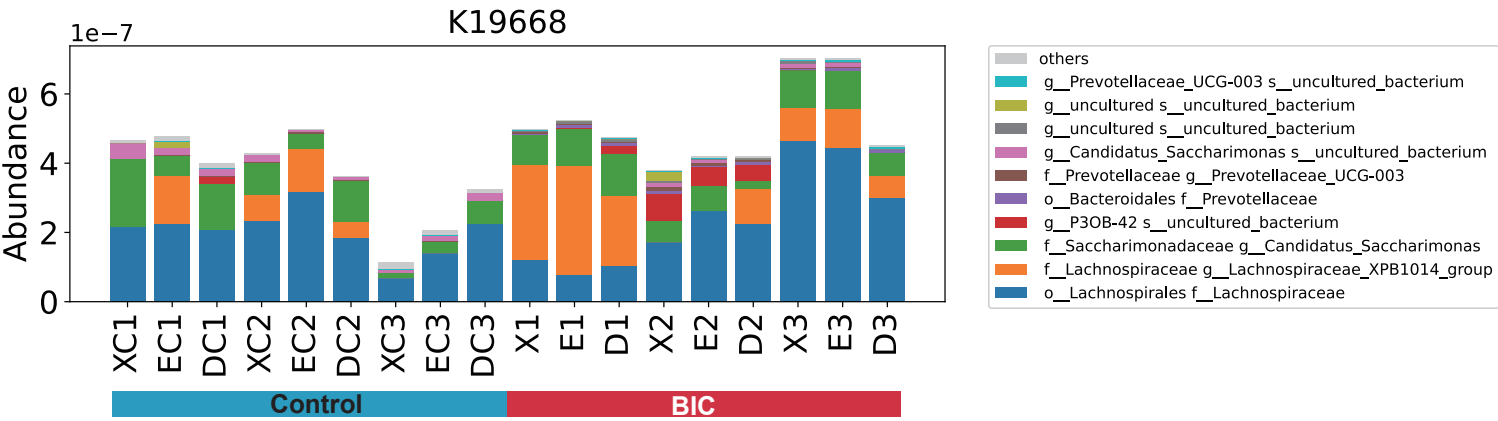

Supplement: Supplementary file 5 — Supplementary Information 5. [file 41598_2025_24196_MOESM5_ESM.pdf]

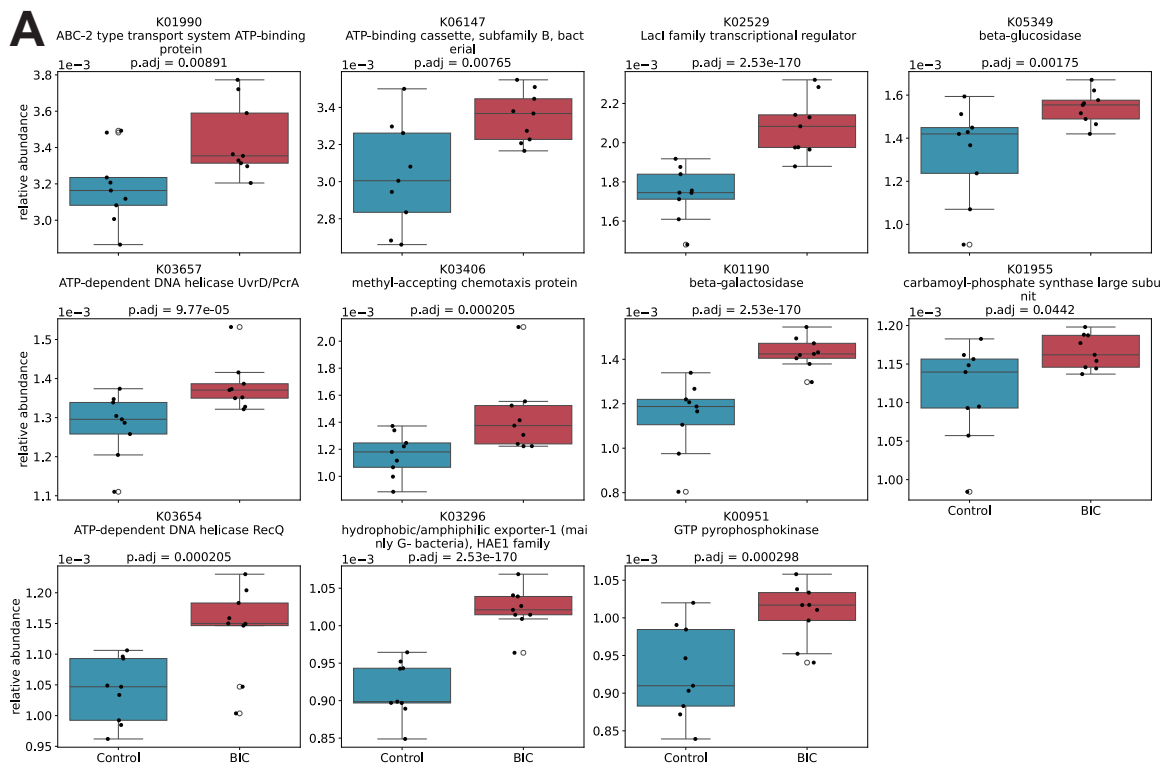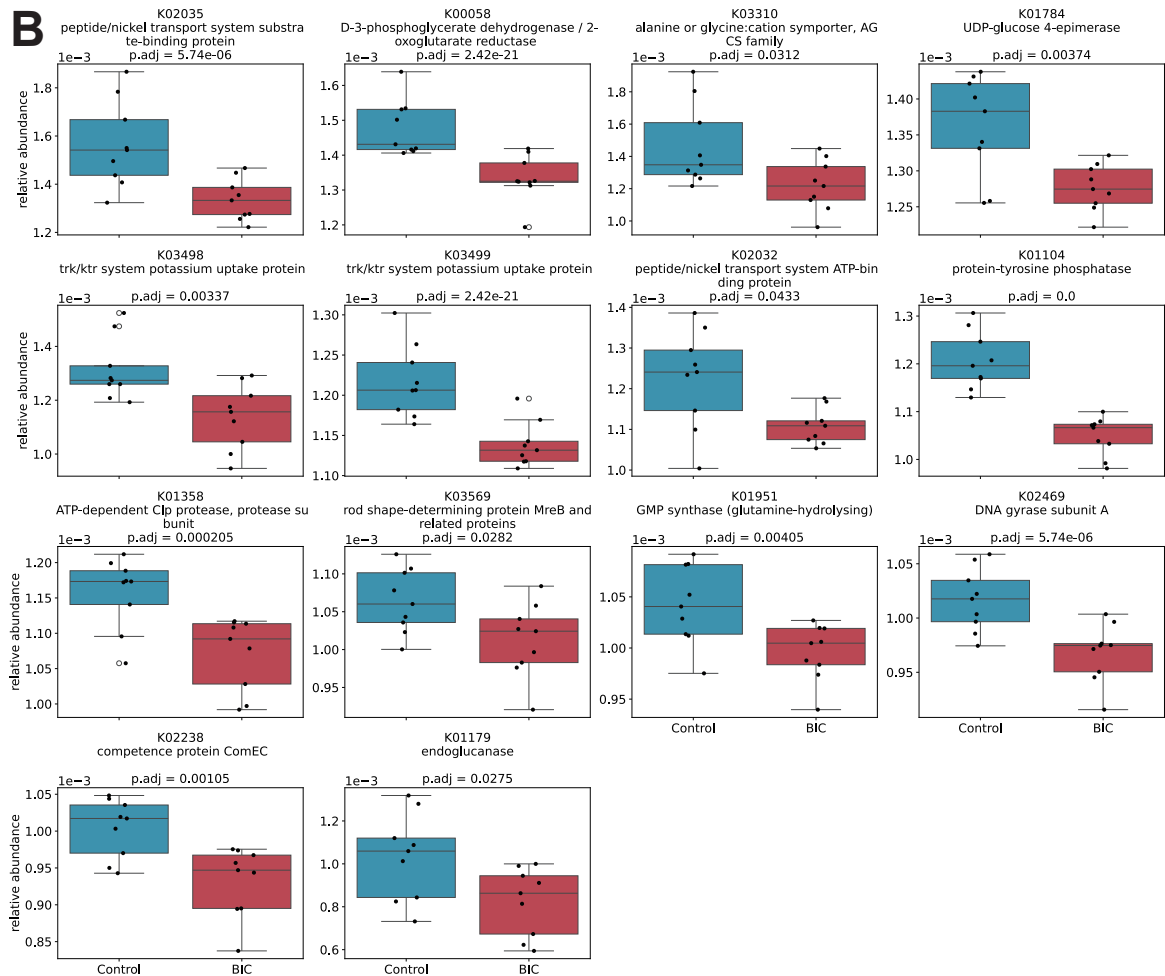

Supplement: Supplementary file 6 — Supplementary Information 6. [file 41598_2025_24196_MOESM6_ESM.pdf]
