# Supplementary material for: Preliminary study of gut microbiome influence on Black Ivory Coffee fermentation in Asian elephants
Source: Sci Rep. 2025 Nov 18;15:40548. doi: 10.1038/s41598-025-24196-0 (PMC12627549; doi:10.1038/s41598-025-24196-0)
Supplement: Supplementary file 7 — Supplementary Information 7. [file 41598_2025_24196_MOESM7_ESM.docx]

**Supplementary Table 1 Results of PERMANOVA.**

Results of a permutational multivariate analysis of variance (PERMANOVA) using Bray–Curtis distances. The items analyzed were as follows: DNA extraction method; classification (BIC elephant or control elephant); age (adult or child, with 10 years as the cutoff); and sex.

**Supplementary Figure S1 Differential analysis of the abundance of individual genera in the gut microbiota.**

The relative abundances of genera that were significantly higher in the BIC elephant group (red) than in the control elephant group (blue). *P* values were calculated by the two-tailed permuted Brunner–Munzel test and adjusted by Benjamini-Hochberg correction. The order of each box plot is based on the mean relative abundance.

**Supplementary Figure S2 KO related to transport pectin-degraded compounds (KdgT transporter, K02526).**

1. KO abundance of K02526. The box plot represents the relative abundance of the KO. The relative abundances of the KO in the BIC elephant group (red) and in the control elephant group (blue). *P* values were calculated by the two-tailed permuted Brunner–Munzel test and adjusted by Benjamini–Hochberg correction.
2. The stacked bar plot represents the bacterial abundance contributing to the KO. The top 10 bacteria with the highest average relative abundance are displayed. Each bar plot represents the abundance of KO from each sample of the control elephant (left side) and the BIC elephant (right side).

**Supplementary Figure S3 Bacterial contribution to the KOs which related to pectin degradation.**

The stacked bar plot represents the bacterial abundance contributing to the KO. The relative abundances of six kinds of bacteria with all genes classified to the 11 KOs are displayed. Each bar plot represents the abundance of KO from each sample of the control elephant (left side) and the BIC elephant (right side).

**Supplementary Figure S4 The number of bacteria that have pectin degradation-related KOs.**

The number of bacteria that have essential KOs to degrade pectin. Depending on which KOs are used, there are five patterns of degradation pathways.

**Supplementary Figure S5 KO related to cellulose degradation.**

1. KO abundance with the cellulose degradation. The box plot represents the relative abundance of the KO. The relative abundances of the KO were significantly higher in the BIC elephant group (red) than in the control elephant group (blue). *P* values were calculated by the two-tailed permuted Brunner–Munzel test and adjusted by Benjamini-Hochberg correction.
2. The stacked bar plot represents the bacterial abundance contributing to the KO. The top 10 bacteria with the highest average relative abundance are displayed. Each bar plot represents the abundance of KO from each sample of the control elephant (left side) and the BIC elephant (right side).

**Supplementary Figure S6 Differential analysis of the abundance of individual KOs of the gut microbiota.**

1. The distribution of relative abundances of KOs was significantly higher in the BIC elephant group (red) than in the control elephant group (blue), and the mean relative abundance was more than 0.001 in all samples. *P* values were calculated by the two-tailed permuted Brunner–Munzel test and adjusted by Benjamini-Hochberg correction.
2. The distribution of relative abundances of KOs was significantly lower in the BIC elephant group (red) than in the control elephant group (blue), and the mean relative abundance was more than 0.001 in all samples. *P* values were calculated by the two-tailed permuted Brunner–Munzel test and adjusted by Benjamini-Hochberg correction.
